# Development of a BM7G(TKO/hCD46/hCD55/hTHBD/hEPCR) donor pig with endogenous promoter-driven transgenes for xenotransplantation

**DOI:** 10.3389/fimmu.2026.1827497

**Published:** 2026-06-23

**Authors:** Cong Xia, Meng Lian, Bingxiu Ma, Hongliang Yu, Renquan Zhang, Lijia Wen, Xueliang Wang, Yu Zhao, Zhen Ouyang, Yinghua Ye, Xiner Feng, Han Wu, Liangxue Lai

**Affiliations:** 1 Institute of Development and Regeneration, Guangzhou Institutes of Biomedicine and Health, Chinese Academy of Sciences, Guangzhou, China; 2 University of Chinese Academy of Sciences, Beijing, China; 3 Guangdong Provincial Key Laboratory of Large Animal Models for Biomedicine, Wuyi University, Jiangmen, China; 4 Key Laboratory of Zoonosis Research, Ministry of Education, College of Animal Science, Jilin University, Changchun, China; 5 Department of Hepatobiliary and Pancreatic Surgery, General Surgery Center, The First Hospital of Jilin University, Changchun, China; 6 Sanya Institute of Swine Resource, Hainan Provincial Research Centre of Laboratory Animals, Sanya, China

**Keywords:** coagulation response, complement regulation, endogenous promoter, multi-genetic modification, xenotransplantation

## Abstract

**Introduction:**

Xenotransplantation holds promise for addressing the organ shortage crisis. Multi-genetic modification of pigs, such as knockout of three carbohydrate antigen-related genes and expression of immunoprotective proteins, can significantly improve xenograft survival. However, existing multi-gene modification strategies face challenges: transposon-based transgenic technology may lead to unstable expression, while exogenous promoters used in site-specific integration strategies are susceptible to epigenetic silencing, making it difficult to maintain long-term, stable expression levels. Therefore, developing a donor pig model capable of achieving stable and long-lasting multi-gene expression is a critical need in the field.

**Methods:**

CRISPR-Cas9 technology was used to knockout three major glycan antigen genes (*GGTA1*, *CMAH*, *β4GalNT2*) to eliminate hyperacute rejection. Subsequently, four human protective genes (hCD55, hCD46, hTHBD, hEPCR) were site-specifically integrated into the porcine *Rosa26* safe-harbor locus. Their expression was driven by the porcine endogenous *Rosa26* promoter and the *THBD* core promoter, respectively, to ensure long-term stable and tissue-specific expression. Furthermore, the selection marker gene was efficiently removed using the Cre/loxP system.

**Results:**

The three glycan antigens were completely absent at both cellular and tissue levels in BM7G genetically modified pigs. What’s more, four protective proteins were stably expressed in vascular endothelial cells and major organs such as the heart, liver, and kidneys. Among them, hCD55 and hCD46 were widely expressed, while hTHBD and hEPCR were specifically expressed in the vascular region. *In-vitro* functional assays confirmed that BM7G porcine vascular endothelial cells significantly reduced the binding of human antibodies, effectively inhibited complement-dependent cytotoxicity, and decreased the formation of thrombin-antithrombin (TAT) complexes.

**Conclusion:**

In summary, by combining the knockout of xenoantigens with the use of endogenous promoters to drive the expression of multiple human protective genes, we successfully constructed a seven-gene modified pig model with low immunogenicity and synergistic protective functions. This provides an important donor resource for preclinical research in xenotransplantation.

## 1 Introduction

Genetically modified pigs represent the most promising source of organs for xenotransplantation to address the global shortage of donor organs (1). Recent years have witnessed critical advances in both preclinical and early clinical studies. Significant progress has been made in life-supporting pig-to-non-human primate (NHP) transplants of hearts, kidneys, and islets (2–4). Furthermore, transplantation studies using brain-dead human decedents have successfully demonstrated short-term renal (5), hepatic (6), and pulmonary (7) xenotransplantation. Notably, initial clinical trials including the transplantation of genetically modified porcine organ into human patients have been conducted (8, 9), marking a critical step toward clinical application.

However, cross-species transplantation triggers vigorous xenogeneic immune rejection, necessitating multi-gene modification of donor pigs as a core strategy (10). Pre-existing natural antibodies in humans recognize carbohydrate xenoantigens, primarily α-Gal, Neu5Gc, and Sda, synthesized by *GGTA1*, *CMAH*, and *β4GalNT2* genes, respectively. Their binding immediately activates the complement cascade, leading to hyperacute rejection (11). Decay-accelerating factor (hCD55) inhibits the complement cascade by accelerating the decay of C3 convertase (12), while membrane cofactor protein (hCD46) acts as a cofactor for the factor I-mediated proteolytic inactivation of C3b and C4b (13). Both effectively inhibit the formation of the membrane attack complex, mitigating complement-mediated injury. Kim et al (14) achieved prolonged graft survival of over 400 days for *GGTA1* KO/hCD55 porcine kidneys in rhesus macaque by employing a regimen that included selective CD4(+) T cell depletion. In a cardiac model, Mohiuddin et al (15) achieved a survival of 236 days for GTKO/hCD46 porcine hearts transplanted into baboons using a CD154-targeted immunosuppressive regimen, validating the efficacy of this modification. Coagulation dysregulation, such as thrombotic microangiopathy and consumptive coagulopathy, presents another major barrier to long-term xenograft survival (16). Molecular incompatibilities in the coagulation pathways are a key cause; for instance, the complex formed between porcine thrombomodulin (THBD) and human thrombin cannot efficiently activate protein C, disrupting the anticoagulant balance (17). *In-vitro* studies (18) have demonstrated that porcine endothelial cells modified with GTKO/hCD55, hCD46, hTHBD, and hEPCR exhibit characteristics similar to human endothelial cells in suppressing human platelet aggregation. Mohiuddin et al (19) have reported achieving a cardiac xenograft survival of up to 945 days in baboons using pigs modified with GTKO/hCD46/hTHBD. In summary, knockout of the three carbohydrate xenoantigens-related genes combined with expression of complement and coagulation regulatory proteins forms a fundamental set of genetic modifications for xenotransplantation donor pigs (20, 21). To date, various genetically modified pigs have been developed (22–26) and applied in the aforementioned studies, driving significant advancements in the field.

Currently, multi-gene modifications in pigs primarily rely on two strategies: transgenic approaches and site-specific knock-in, each presenting distinct challenges. Although the PiggyBac transposon-based transgenic technology enables efficient integration, random insertion may lead to unstable expression and copy number variations in offspring (27), affecting the reliable inheritance of phenotypes. Site-specific knock-in strategies based on homology-directed repair (HDR) (28) or recombinase-mediated cassette exchange (RMCE) (29, 30) allow precise integration of the target gene into predetermined genomic loci (e.g., safe harbor sites (31)), which significantly improves the genetic stability of the transgene. However, even with targeted integration, the exogenous promoters used may still be susceptible to host epigenetic silencing mechanisms (32, 33), leading to gradual attenuation of transgene expression and making it difficult to maintain long-term, stable expression levels *in-vivo (*34).

Using endogenous promoters to drive transgenes is an effective strategy for achieving long-term expression (35). According to Li et al., the endogenous promoter of the porcine *Rosa26* (*pRosa26*) safe harbor locus exhibits high expression activity in various organs (e.g., liver, kidney, heart) (36). Similarly, the porcine thrombomodulin (*pTHBD*) promoter efficiently drives the expression of *pTHBD* in porcine endothelial cell lines, making it suitable for driving human THBD expression, with stable inheritance to offspring (37). In this study, we report the generation of a seven-gene-modified Bama miniature pig model. Three glycoantigen-related genes were knocked out, while four human protective genes were inserted into the *pRosa26* locus. Specifically, the complement regulators hCD55 and hCD46 are expressed under the control of the endogenous *pRosa26* promoter, whereas the coagulation regulators hTHBD and hEPCR are regulated by the *pTHBD* core promoter. This strategy effectively avoids​ the common issue of epigenetic silencing associated with exogenous promoters by utilizing endogenous promoters, thereby providing a reliable solution for generating multi-genetically modified pig models with stable and long-term transgene expression.

## 2 Methods

### 2.1 Pig welfare

Chinese Bama miniature pigs were used for the experiment. Animal studies were conducted with the approval of Animal Ethic Committee of the South China Institute of Large Animal Model for Biomedicine (Wuyi-2026-02). The pigs were housed in a facility under standard environmental conditions, including controlled temperature, humidity and a 12 h light/dark shift. Healthy and physiological condition were checked daily by veterinarians.

The euthanasia of pigs was performed by exsanguination. Prior to exsanguination, the pigs were sedated via intramuscular injection of Zoletil 50 (5 mg/kg). The depth of anesthesia was assessed by applying mechanical stimulation to the toes of the pig. The absence of withdrawal reflexes was used to confirm that the animal had reached an adequate depth of anesthesia.

### 2.2 Generation of guide RNA plasmid for triple knockout

The sequences of the target genes *GGTA1*, *β4GalNT2*, and *CMAH* were download from the NCBI database. Guide RNAs (gRNAs) were designed using the online software (https://portals.broadinstitute.org/gppx/crispick/public). gRNA-specific primers were synthesized by IGEbio Genomics Co., Ltd. Specifically, the *GGTA1* gene (NCBI Gene ID: 396733) was targeted at exon 6 with the gRNA sequence 5’-TCATGGTGGATGATATCTCCAGG-3’, while *CMAH* (NCBI Gene ID: 396918) was targeted at exon 1 with the gRNA sequence 5’-GAAGCTGCCAATCTCAAGGAAGG-3’. For *β4GalNT2* (NCBI Gene ID: 100621328) a single gRNA was designed to simultaneously knock out exon 10 of both genes, with the sequence 5’-GGATGGCTTCCCCGACTGCGTGG-3’. Subsequently, the three gRNAs were subcloned into lentiCRISPR v2 (Addgene, Cat. #52961) plasmid.

### 2.3 Assembly of *pRosa26* HDR constructs

For *pRosa26* site-specific insertion, a homologous recombination repair (HDR) template vector was constructed. A 1.2 kb left homology arm and a 1 kb right homology arm were amplified from genomic DNA isolated from Bama miniature pigs. The human coding DNA sequences (CDSs) of human CD55 (hCD55), human CD46 (hCD46), human thrombomodulin (hTHBD), and human endothelial protein C receptor (hEPCR) were synthesized by IGEbio Genomics Co., Ltd. Two polycistronic transcription units were arranged in opposite orientations: one contains a viral splice acceptor (SA), a promoterless hCD55 CDS, and a hCD46 CDS, which are linked by a viral 2A peptide sequence; the other, in the inverted orientation, comprises an hTHBD CDS and hEPCR CDS, driven by the *pTHBD* core promoter (amplified from Bama miniature pig genomic DNA).

### 2.4 Transfection and selection of positive cell colonies

PFFs were cultured and electroporated with the targeting plasmid using the Neon system (1350 V, 30 ms, 1 pulse). Twenty-four hours later, cells were plated at low density and selected with puromycin (1000 ng/mL) for 14 days. For knockout validation, genomic DNA was extracted from expanded clonal lines. The sgRNA-targeted regions were amplified by PCR and subjected to Sanger sequencing, with the resulting sequences compared to wild-type to confirm the genotype. Integration at the *pRosa26* locus was verified using a separate PCR strategy: for the 5’ junction, primers were designed upstream of the 5’ homologous arm and within the splice acceptor (SA) sequence; for the 3’ junction, primers were placed after the puromycin resistance gene polyA tail and downstream of the 3’ homologous arm. To simultaneously detect knock im (KI)and wild-type (WT) alleles, a competitive PCR assay was performed using three primers (Rosa26-KI/WT-F, pRosa-WT-R, and pRosa-KI-R). Biallelic integration yielded only the KI band, heterozygous integration produced both KI and WT bands, and absence of integration yielded only the WT band. This approach ensured that a PCR product was generated only upon correct integration of the full expression cassette. All genotyping primers are listed in **Supplementary Table 4**.

### 2.5 Production of clone piglets

Gene-edited cells were cloned into pigs by SCNT, and cloning was performed as previously described (38–40). Porcine oocytes were enucleated, injected with donor cells, and activated by electrofusion. Reconstructed embryos were cultured briefly and then surgically transferred into synchronized surrogate sows. Cloned piglets were ultimately delivered via cesarean section. The cloned piglets were delivered by cesarean section in DPF facility.

### 2.6 Isolation of porcine peripheral blood mononuclear cells

Fresh anticoagulated whole blood was collected and mixed with an equal volume of PBS. An equal volume of Ficoll-Paque (GE Healthcare, Cat. #17-1440-03) was added into a centrifuge tube, and the diluted blood was slowly layered onto the surface of the separation medium while maintaining a clear interface. After centrifugation at 400×g for 30 minutes, the solution separated into distinct layers. The PBMC fraction layer was carefully aspirated with a pipette and transferred to a new centrifuge tube. The harvested cells were washed with PBS, subjected to erythrocyte lysis, and then counted.

### 2.7 Flow cytometric phenotyping of PBMCs

PBMCs from wild-type (WT) and BM7G pigs were isolated using Ficoll-Paque Plus (GE Healthcare, Cat. #17-1440-03) according to the manufacturer’s protocol. Each sample (100,000 cells per sample) stained with primary and secondary antibodies. aGal was stained by FITC conjugated Isolectin B4 (IB4, SIGMA, Cat. #L2895, 1:100 dilution). β4GalNT2 phenotype was carried out using Fluorescein Dolichos Biflorus Agglutinin (DBA, Cat. Vector Laboratories, FL-1031, 1:50 dilution). The expression level of Neu5Gc was assessed by chicken anti-Neu5Gc antibody (BioLegend, Cat. #146901, 1:300 dilution), the secondary antibody was Alexa Fluor568 goat anti-chicken (Abcam, Cat. #ab96947, 1:1000 dilution), and the chicken IgY Isotype was used as negative control (BioLegend, Cat. #402101). Samples were washed and analyzed on a flow cytometer (CytoFLEX, Beckman). The data were collected and analyzed using FlowJo software (Flowjo_v10.8).

### 2.8 Antibody binding assay

Human sera were obtained from de-identified remnant clinical laboratory samples with approval. Prior to use, all serum samples were inactivated at 56 °C for 30 minutes to eliminate complement activity. PBMCs from WT, TKO, and BM7G pigs were isolated using Ficoll-Paque Plus (GE Healthcare, Cat. #17-1440-03) according to the manufacturer’s protocol. Each sample (100,000 cells per sample) were incubated separately with inactivated 40% human serum from each of the 28 individual donors for 30 min at 4 °C, then washed three times with PBS and blocked with 5% BSA. Subsequently, the cells were stained with goat anti-human IgG Alexa Fluor 488 (Invitrogen, Cat. #A11013; 1:200 dilution) and goat anti-human IgM Alexa Fluor 647 (Invitrogen, Cat. #A21249; 1:200 dilution) for 30 min at room temperature. After three additional washes with PBS, flow cytometric analysis was performed using a CytoFLEX flow cytometer, and data were analyzed with FlowJo software (Flowjo_v10.8).

### 2.9 Detection of transgenic protein expression levels in tissues by western blot

Heart, liver, and kidney tissues from BM7G and TKO pigs were homogenized, and proteins were extracted. Protein concentrations were determined by BCA assay. Western blot was performed using standard electrophoresis, transfer, blocking, and ECL detection procedures. The expression of hCD55, hCD46, hTHBD, and hEPCR was detected using the following primary antibodies: Anti-CD55 Antibody (Bioster, Cat. #BM5281, 1:1000 dilution), Anti-CD46 antibody (Abcam, Cat. #AB108307, 1:1000 dilution), Anti-Thrombomodulin antibody (Abcam, Cat. #AB6980, 1:1000 dilution), and Anti-EPCR/CD201 antibody (Abcam, Cat. #AB236517, 1:1000 dilution). The corresponding secondary antibodies used were Goat Anti-Mouse IgG H&L (HRP) (Abcam, Cat. #ab6789, 1:5000 dilution) and Goat Anti-Rabbit IgG H&L (HRP) (Abcam, Cat. #ab6789, 1:5000 dilution).

### 2.10 Detection of transgenic proteins on porcine cells by immunohistochemistry

Tissue sections were deparaffinized, underwent antigen retrieval, and were blocked with 3% BSA. The sections were incubited with appropriately diluted primary antibodies at 4 °C overnight. After washing with PBS, the corresponding HRP-conjugated secondary antibodies were applied and incubated at room temperature for 1 hour. The following primary antibodies were used: Anti-CD55 Antibody (Bioster, Cat. #BM5281, 1:300 dilution), Anti-CD46 antibody (Abcam, Cat. #AB108307, 1:300 dilution), Anti-Thrombomodulin antibody (Abcam, Cat. #AB6980, 1:300 dilution), and Anti-EPCR/CD201 antibody (Abcam, Cat. #AB236517, 1:300 dilution). The corresponding secondary antibodies were Goat Anti-Mouse IgG H&L (HRP) (Abcam, Cat. #ab6789, 1:1000 dilution) and Goat Anti-Rabbit IgG H&L (HRP) (Abcam, Cat. #ab6789, 1:1000 dilution). Following DAB development, nuclei were counterstained with hematoxylin. The sections were subsequently dehydrated through a graded ethanol series, cleared in xylene, and mounted with neutral resin. All sections were observed and imaged under a bright-field microscope.

### 2.11 Detection of surface antigens and transgenic proteins on porcine cells by immunofluorescence

Tissue sections were deparaffinized, underwent antigen retrieval, and were blocked with 3% BSA. After overnight primary antibody incubation at 4 °C and PBS washes, sections were incubated with fluorescent secondary antibody for 1h in the dark, followed by DAPI nuclear staining. Images were captured using fluorescence microscopy. Following final washes, the sections were observed and images were captured using a fluorescence microscope. The primary antibodies used were as describe above, same as used in Western Blotting. The corresponding secondary antibodies used were Goat Anti-Mouse IgG H&L (Alexa Fluor^®^ 488) (Abcam, Cat. #ab150113, 1:1000 dilution) and Goat Anti-Rabbit IgG H&L (Alexa Fluor^®^ 594) (Abcam, Cat. #ab150080, 1:1000 dilution).

### 2.12 Isolation of porcine vascular endothelial cells (PECs)

Following euthanasia of pigs, the aortas were aseptically isolated and transferred into 150-mm sterile culture dishes. The specimens were rinsed repeatedly with sterile PBS, and redundant connective and adipose tissues surrounding the vessels were completely removed. The aortas were gently everted to fully expose the endothelial surfaces, followed by digestion with collagenase type IV (Sigma-Aldrich, Cat. #G5138) at 38°C for 2 h. After the digestion was terminated with stop buffer, porcine vascular endothelial cells (PECs) were gently scraped 8 times in a single direction using a sterile cell scraper. All washing suspensions were collected, and the cells were harvested by centrifugation and cryopreserved separately. The isolated cells were stained with PECAM-1 Alexa Fluor^®^ 488-conjugated Antibody (R&D Systems, Cat. #FAB33871G-100ug) to determine their purity.

### 2.13 Complement-dependent cytotoxicity assay

WT, TKO, and BM7G endothelial cells (100,000 cells per sample) were incubated separately with 40% human serum from each of the 10 individual donors at 37°C for 30 min. After incubation, the cells were stained with propidium iodide (PI). After 3 minutes, the percentage of PI-positive cells was detected using a CytoFLEX flow cytometer. The PI-positive rate of the serum-free control group for each cell type was quantified as the background value. Each data point was then subtracted by its corresponding background value to obtain the corrected cell death rate.

### 2.14 TAT formation assay

WT, TKO, and BM7G endothelial cells were seeded in 24-well plates at a density of 300,000 cells per well. After 24 hours of culture, the cells were incubated with fresh human whole blood collected in sodium citrate anti-coagulant tube. Following incubation at 37 °C with gentle shaking for 1 hour, the plasma was separated. The concentration of the thrombin-antithrombin (TAT) complex in the plasma was measured using a Human Thrombin–Antithrombin Complex ELISA Kit (ab108907, Cat. # Abcam).

### 2.15 Statistics analysis

Statistical analysis was performed using GraphPad Prism 8 software. A two-tailed Student’s t-test or ANOVA was used for comparisons, and statistical significance was defined as a P value of < 0.05.

## 3 Results

### 3.1 Production of BM7G donor pigs

**Figure 1:**
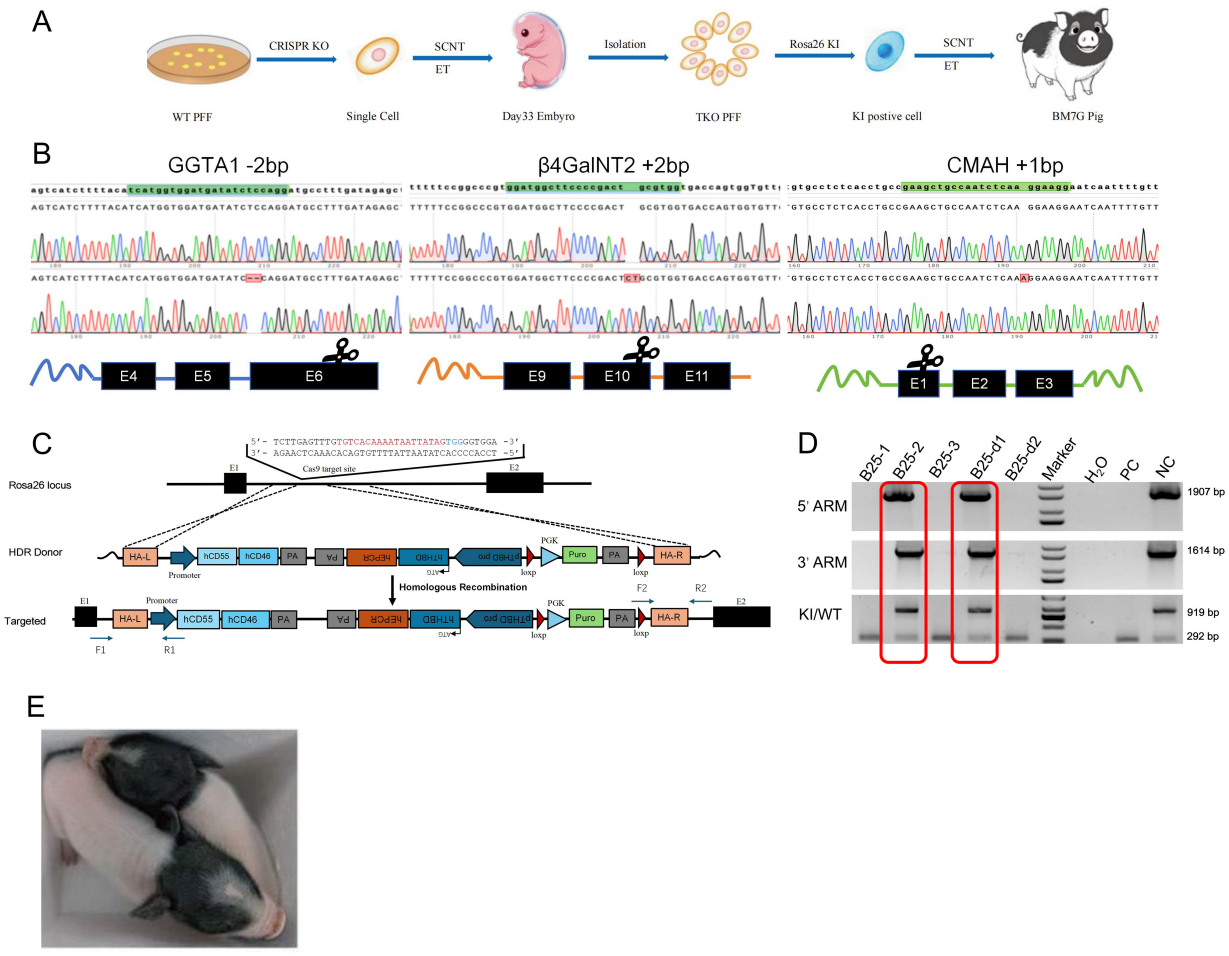
Generation of BM7G pigs for xenotransplantation. **(A)** The workflow for generating BM7G pigs via continuous cloning and multiple rounds of gene editing. **(B)** Sanger sequencing results of *GGTA1* (targeting exon 6), *CMAH* (targeting exon 1), and *β4GalNT2* (targeting exon 10) in WT and Triple-KO positive fetuses. **(C)** Strategy for targeted knock-in of a dual transgene expression cassette into the porcine *Rosa26* locus. **(D)** Genotyping of piglets by PCR analysis. Piglets B25–2 and B25-d2 tested positive for both the 5’ (1382 bp) and 3’ (1614 bp) homology arms of the targeted *Rosa26* locus. The concurrent presence of both knock-in (KI) and wild-type (WT) bands indicates a monoallelic knock-in​ event **(E)** BM7G-positive cloned piglets.

The engineering workflow was presented in **Figure 1A**. Firstly, Cas9 expression plasmid together with sgRNAs targeting the *GGTA1*, *CMAH*, and *β4GalNT2* genes was delivered into wild-type (WT) Bama PFF cells via electroporation (**Supplementary Table 1**). Triple-gene knockout (TKO) cell clones were obtained and used for somatic cell nuclear transfer (SCNT) and embryo transfer. A pregnant surrogate was sacrificed at day 33 after the embryo transfer. Twenty fetuses were collected to isolate PFF cells, genotyped results indicated 10 of these fetuses were biallelic TKO (numbers: 9#-14#, 16#, 17#, 18#, 20#) (**Figure 1B**) (**Supplementary Table 2**). Then, a HDR knock-in vector targeting the *pRosa26* locus was constructed, which contained two expression cassettes to enable the expression of four immune-protective proteins (hCD55, hCD46, hTHBD, and hEPCR) (**Figure 1C**). This HDR donor vector, together with a Cas9 and *pRosa26*-sgRNA plasmid, were electroporated into TKO PFF cells. After selection with puromycin, positive *pRosa26* knock-in cell clones were obtained, which were confirmed to be correctly targeted by 5′- and 3′-arm PCR analysis (**Supplementary Figure 1**).

Subsequently, SCNT and embryo transfer were performed using these positive cells as nuclear donor. The 2 surrogate sows gave birth to 5 cloned piglets. Genotyping confirmed that two of the piglets carried a monoallelic​ knock−in at the *pRosa26* locus (**Figure 1D**), with a genotype of TKO/hCD55/hCD46/hTHBD/hEPCR. These animals were designated as BM7G (**Figure 1E**).

### 3.2 Cre/loxP-mediated excision of the selection marker

**Figure 2:**
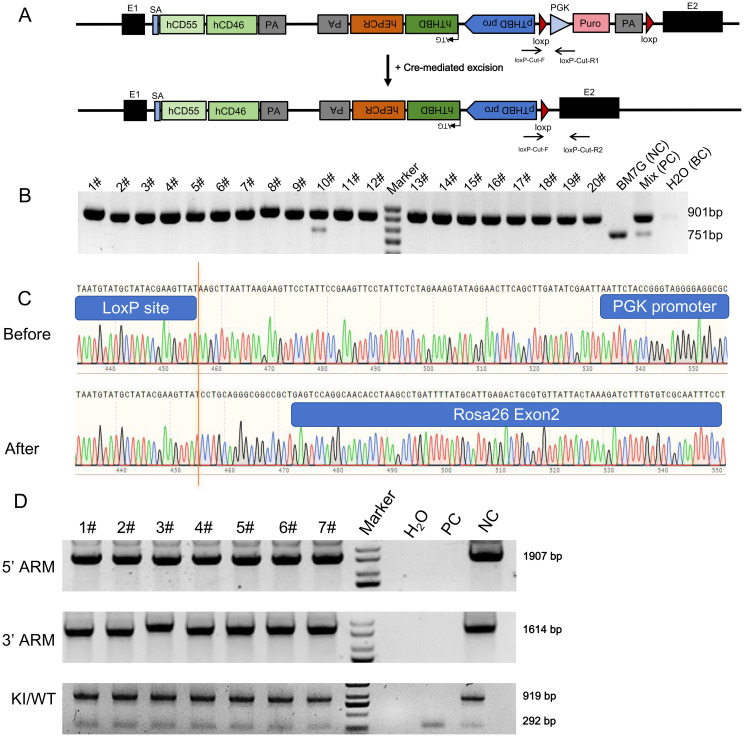
Cre-mediated deletion of selection marker. **(A)** Strategy for the deletion of the Selection Marker. **(B)** Genotyping of cell clones by PCR analysis. Untransfected cells (BM7G (NC)) served as the negative control, and a mix of cells collected during the experiment was used as the positive control. The specific bands at 901 bp and 751 bp confirm the precise deletion event. **(C)** Comparison of Sanger sequencing chromatograms for the recombination locus. The lower panel (After) clearly shows the sequence junction after Cre recombinase treatment. **(D)** Genotyping of Cloned Pig Offspring by PCR. Lanes 1-7: Seven different cloned pig samples; H_2_O: no-template control (water); PC: Positive control (genomic DNA from BM7G pig); NC: Negative control (genomic DNA from wild-type pig). KI/WT: Knock In/Wild Type.

To obtain genetically modified cells without a selection marker, ear tissue was collected from BM7G donor pigs to isolate primary ear fibroblasts. Next, transient transfection of a Cre-expressing plasmid was performed to mediate the deletion of the selection marker between the two loxP sites (**Figure 2A**). A total of 20 cell clones were picked and subjected to PCR genotyping. Gel electrophoresis results showed that, with the exception of clone 10#, the PGK-Puro selection cassette was successfully excised in the remaining 19 cell clones (**Figure 2B**), representing an excision with high efficiency. Sanger sequencing further confirmed the precise removal of the selection marker, leaving only a single loxP (**Figure 2C**). Then, the positive cells were used as nuclear donors for SCNT. A total of 676 embryos were transferred into 4 surrogates. Ultrasound examination one month later revealed that 3 of the sows were pregnant. The pregnant sows were maintained under specific pathogen-free conditions and finally delivered by cesarean section, yielding 12 piglets, 7 of which survived (**Supplementary Table 3**). Genomic DNA PCR analysis of the surviving piglets confirmed that all possessed a monoallelic​ knock-in at the *pRosa26* locus and were derived from the aforementioned SCNT donor cells (**Figure 2D**).

### 3.3 Detection of protein expression at the cellular level

**Figure 3:**
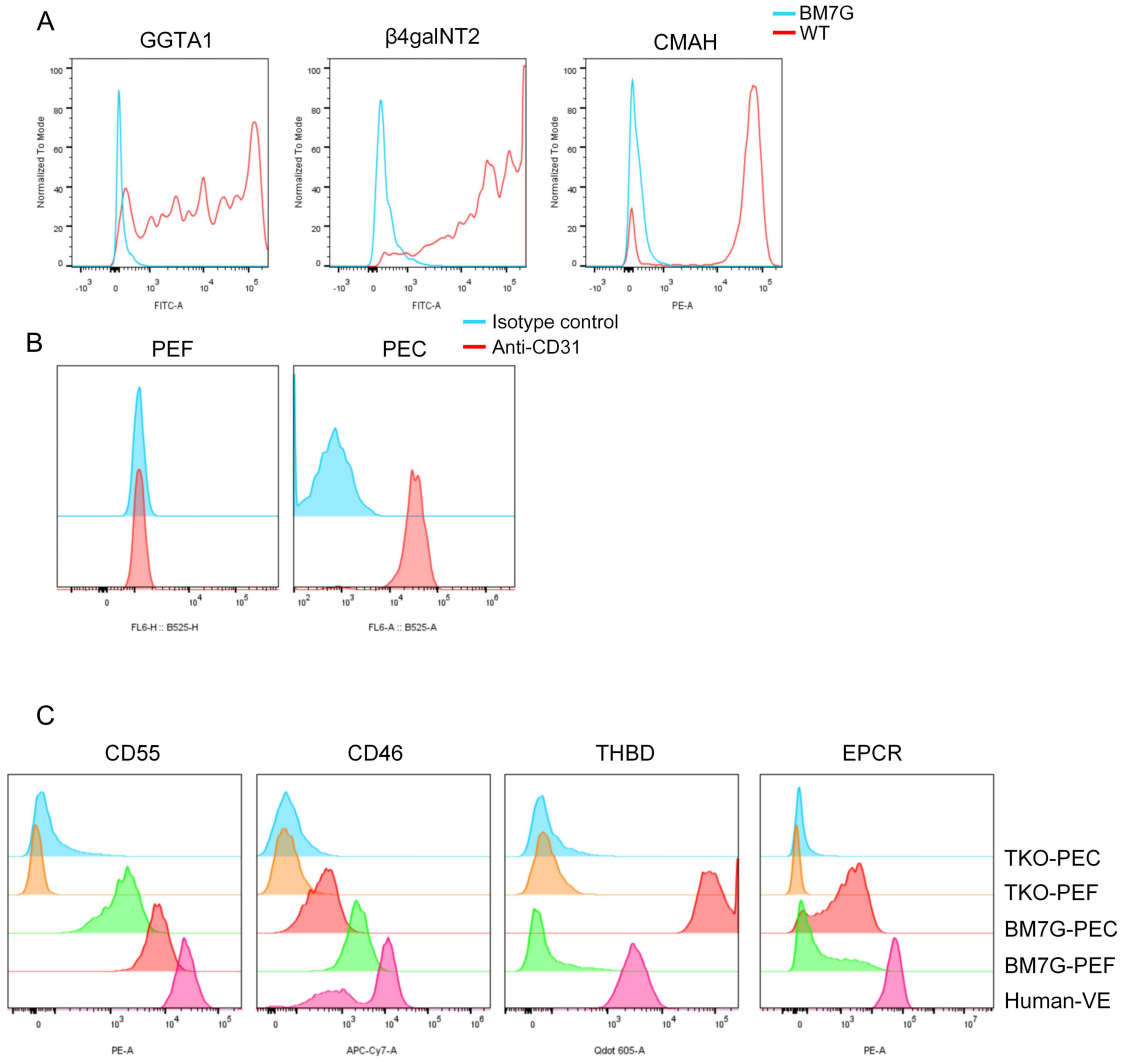
Detection of protein expression at the cellular level. **(A)** Comparison of α-Gal (*GGTA1*), SDa (*β4GalNT2*), and Neu5Gc (*CMAH*) expression on PBMCs from BM7G (blue) and WT (red) pigs. **(B)** Detection of the endothelial marker CD31 on PEFs and PECs. **(C)** Flow cytometric analysis of four immune-protective proteins in TKO-PEC, TKO-PEF, BM7G-PEC, BM7G-PEF, Human-VE.

To examine whether three xenoantigens (α-Gal, Sda, and Neu5Gc) were completely eliminated, peripheral blood mononuclear cells (PBMCs) were isolated from the WT and BM7G pigs. Subsequently, the PBMCs were stained with fluorescently labeled antibody, respectively. Flow cytometry analysis of PBMCs revealed complete absence of α-Gal, Sda, and Neu5Gc antigens in BM7G pigs (**Figure 3A**), consistent with functional knockout of *GGTA1*, *CMAH*, and *β4GalNT2* (**Figure 1B**).

While knocking out the three genes responsible for synthesizing these glycan epitopes, four immune-protective protein genes were integrated into the *pRosa26* locus. We assessed the expression patterns of these transgenes across distinct cell types of BM7G pigs. TKO vascular endothelial cells (TKO-PEC), BM7G vascular endothelial cells (BM7G-PEC), and BM7G ear fibroblasts (BM7G-PEF) were isolated and cultured (**Figure 3B**). Then the transgene expression across different cell lines were analyzed by flow cytometry, with human vascular endothelial (VE) cells used as a control. Compared to TKO-PEC, BM7G-PEC exhibited significantly elevated expression of hCD55, hCD46, hTHBD, and hEPCR (**Figure 3C**). In contrast, BM7G-PEF expressed only hCD55 and hCD46, consistent with the ubiquitous activity of the *pRosa26* promoter. This expression pattern further confirms the endothelium-specific activity of the *pTHBD* promoter. Notably, hCD55 and hCD46 expression levels in BM7G endothelial cells were comparable to those observed in human VE cells, whereas hTHBD expression was markedly higher.

### 3.4 Detection of protein expression at the organ level

**Figure 4:**
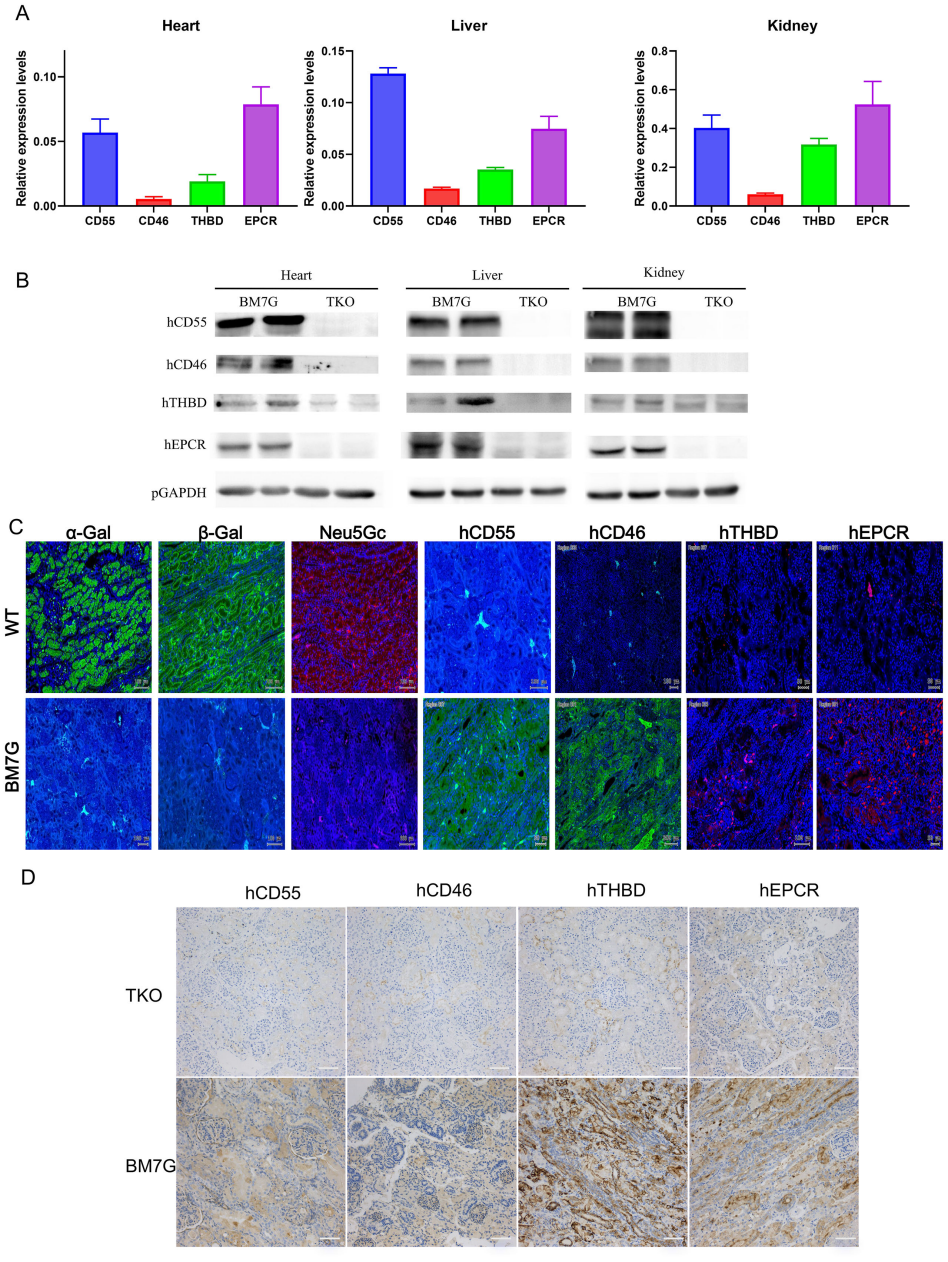
Detection of protein expression at the organ level. **(A)** RT-qPCR analysis of immune-protective gene expression in heart, liver, and kidney. **(B)** Western Blot analysis of immune-protective gene expression in heart, liver, and kidney. **(C)** Immunofluorescence staining of kidney sections comparing WT and BM7G pigs. Scale bar = 100um. **(D)** Immunohistochemical staining of human protective proteins in kidney sections comparing TKO and BM7G pigs. Scale bar = 100um.

To further verify the expression of these four proteins at the organ level, heart, liver, and kidney tissues were collected from the BM7G pig and performed RT-qPCR and Western blot analysis. At both the mRNA and protein levels, expression of the four immune-protective proteins was detected in all three tissues (**Figures 4A, B**). We also characterized the genetic modifications in heart, liver, and kidney tissues via immunofluorescence staining for the three glycan epitopes and four immune-protective proteins. Compared to the organ tissues from WT pig, α-Gal, Sda, and Neu5Gc antigens were completely negative in the heart, liver, and kidney tissues of BM7G pigs (**Figure 4C**, **Supplementary Figure 2**). All four transgenic proteins were successfully detected in the heart, liver, and kidney tissues of BM7G pigs. The complement regulatory proteins, hCD55 and hCD46, were prominently expressed and widely distributed, while the coagulation regulatory proteins hTHBD and hEPCR were specifically localized to the vascular regions of these tissues (**Figure 4C**, **Supplementary Figure 2**). This specific expression pattern indicates that the endogenous *pRosa26* promoter drives stable and ubiquitous expression of complement regulate proteins, whereas the *pTHBD* promoter exhibits strong endothelial specificity. Furthermore, immunohistochemical staining was performed to verify the expression of hCD55, hCD46, hTHBD, and hEPCR in the three organs of BM7G pigs (**Figure 4D**, **Supplementary Figure 3**). The results further confirmed stable expression of these four protective proteins at the organ level.

### 3.5 Immunogenicity detection of BM7G

**Figure 5:**
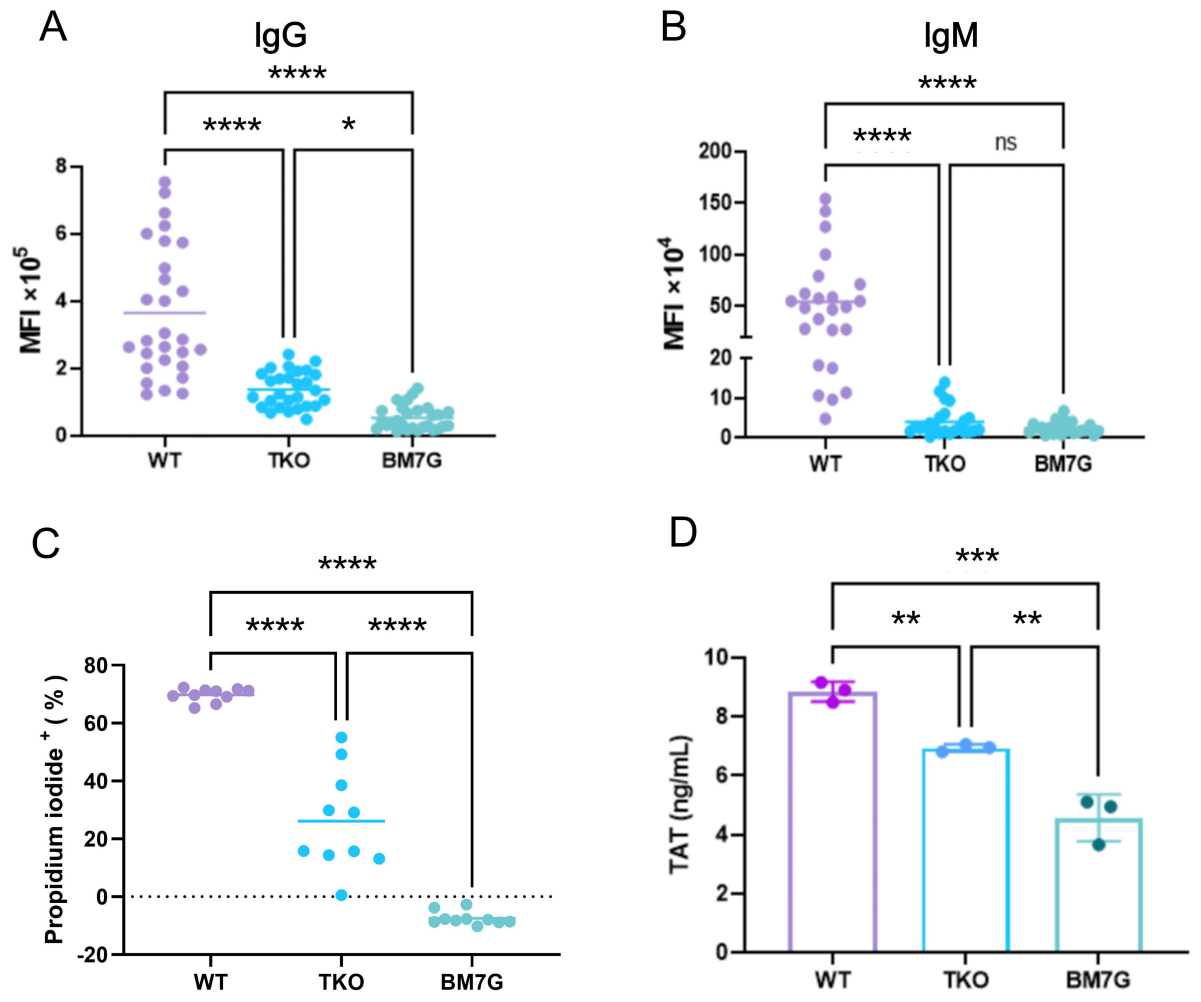
Immunogenicity detection of BM7G. Antibody binding assay: Flow cytometric analysis of human IgG **(A)** and IgM **(B)** binding to PBMCs isolated from WT, TKO, and BM7G pigs. **(C)** Complement-dependent cytotoxicity (CDC) assay on porcine vascular endothelial cells. **(D)** Thrombin-antithrombin (TAT) complex generation assay using porcine vascular endothelial cells. Statistical significance was determined by one-way ANOVA (****, p < 0.0001, ***, p < 0.001, **, p<0.01, *, p < 0.05). Data are shown as mean ± SD.

After confirming the negative status of the three glycan epitopes, the immunogenicity of BM7G pig cells was evaluated by incubating PBMCs from WT, TKO, and BM7G pigs with 40% heat-inactivated human serum. Significantly reduced binding of human IgM and IgG antibodies was observed in both BM7G and TKO PBMCs compared to WT controls, indicating that knockout of the *GGTA1*, *β4GalNT2*, and *CMAH* genes greatly diminished xenograft immunogenicity (**Figures 5A, B**). To further assess the function of the expressed complement regulatory proteins, CDC assays were performed using endothelial cells isolated from the pigs. The results showed that the TKO group significantly reduced complement-mediated cytotoxicity compared to the WT group. The positive rate of PI of the BM7G group were further reduced to nearly zero after background subtraction, demonstrating the functional activity of the expressed hCD55 and hCD46 (**Figure 5C**). Finally, the anti-coagulant functions of hTHBD and hEPCR were verified via a thrombin-antithrombin complex (TAT) assay, in which PEC from BM7G co-cultured with fresh human whole blood significantly reduced TAT formation compared to WT and TKO cells, indicating effective regulation of coagulation and potential alleviation of coagulation disorders in xenotransplantation (**Figure 5D**).

### 3.6 BM7G off-target assessment

**Figure 6:**
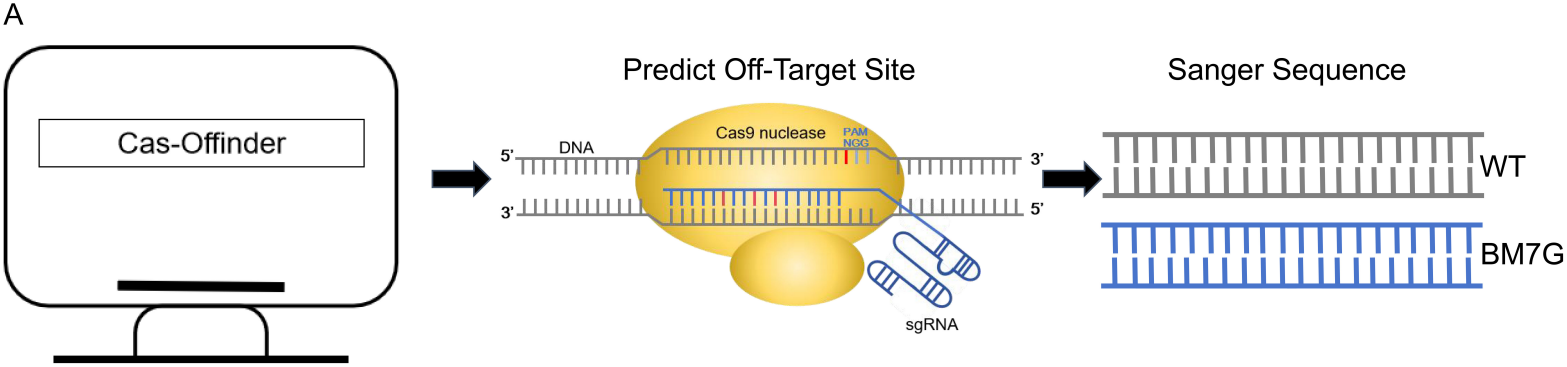
BM7G off-target assessment. Potential off-target sites (≤3 bp mismatches) were predicted using Cas-OFFinder, and the corresponding regions in wild-type (WT) and BM7G samples were subjected to Sanger sequencing for validation.

To test whether off-targeting occurred in BM7G pig, potential off-target site of *GGTA1*, *CMAH*, *β4GalNT2* and *pRosa26* sgRNA were screened using Cas-OFFinder (http://www.rgenome.net/cas-offinder/) (41). We amplified these off-target sites from BM7G and WT pig genome by PCR and performed Sanger sequence (**Figure 6**). These results indicated that genome editing occurred in only the targeted region, and no off-target mutations were introduced at other untargeted sites (**Table 1**, **Supplementary Figure 4**).

**Table 1:** BM7G off-target assessment.

Gene	*GGTA1*	*β4GalNT2*	*CMAH*	*Rosa26*
Number of predicted sites	9	8	9	10
Number of off-target sites	0	0	0	0

## 4 Discussion

In this study, we successfully developed a novel seven-gene-modified xenotransplantation donor pig model. Its construction was based on two core genetic engineering strategies: first, the three major xenoantigen genes (*GGTA1*, *β4GalNT2*, and *CMAH*) were completely knocked out to eliminate hyperacute rejection; second, four human protective genes were site-specifically integrated into the *pRosa26* safe harbor locus. By utilizing endogenous promoters, these genes were stably expressed in various organs such as the heart, liver, and kidneys, driving the expression of human complement regulatory proteins (hCD55 and hCD46) and thrombomodulins (hTHBD and hEPCR) to simultaneously suppress complement activation and coagulation dysregulation (42, 43). To evaluate their function, systematic *in-vitro* validation was performed. The results showed that PEC derived from BM7G pigs significantly alleviated pre-existing antibody-mediated immune rejection and effectively inhibited coagulation pathway activation, demonstrating that the multiple modifications exert synergistic protective effects at the cellular level.

Rosa26 is widely expressed in both embryonic and adult tissues (44). Targeting genes to the Rosa26 locus represents an ideal method for creating transgenic animals with sustained and high-level transgene expression (45, 46). Li et al (36) successfully generated *pRosa26*-targeted pigs and observed widespread reporter gene expression at both fetal and adult stages, thereby validating the general applicability of this locus in pig models. However, in the field of xenotransplantation, when Fang et al (47) integrated expression cassettes containing hCD55 and hCD47 genes driven by an IHK chimeric promoter into the *pRosa26* locus, an low level expression of these two proteins was detected at the erythrocyte or platelet level. This was likely due to epigenetically mediated transcriptional silencing or differences between human and porcine transcriptional mechanisms (48, 49). Subsequent experiments in PK−15 cell lines further demonstrated that using the porcine endogenous *CD47* promoter could improve the expression levels of CD55 and CD47, suggesting that endogenous promoters play a crucial role in driving stable expression of exogenous immune-protective genes. Furthermore, the long-term expression of exogenous proteins is a critical factor for prolonged survival in xenotransplantation. Wang et al (50) successfully inserted a Cre-dependent Cas9 expression system into the *pRosa26* locus and achieved rapid development of lung tumors in pigs by delivering Cre recombinase and sgRNA. Building on this, Jin et al (51) integrated a Tet-On system for inducible expression of KRASG12D into the *pRosa26* locus and successfully observed squamous cell carcinoma phenotypes in the nose, mouse and scrotum of F1-generation pigs. Therefore, in this study, all human protective genes were site-specifically integrated into the *pRosa26* locus and driven by porcine endogenous promoters rather than traditional exogenous viral promoters, to achieve stable and long-term expression of the immunoprotective genes.

The presence of a selection marker gene can limit subsequent genetic manipulations within the same individual, and the currently available types of selection markers are limited (52). Therefore, its removal via the Cre/loxP system facilitates the future cultivation of donor pigs with more complex genetic modifications. At present, the elimination of selection markers has not received sufficient attention in the field of xenotransplantation. However, in the production of recombinant protein drugs, residual selection marker genes and their expression products may increase the immunogenicity risk. This approach not only eliminates associated safety and regulatory concerns but also lays the foundation for further model optimization to ultimately meet stringent clinical application requirements.

Recent clinical trials in xenotransplantation have achieved milestone breakthroughs. A report from Massachusetts General Hospital documented a 69-gene-edited pig kidney transplanted into a human surviving for 271 days, while Xijing Hospital reported a 261-day survival for a kidney from a 6-gene-edited pig. Additionally, in a groundbreaking case of auxiliary liver xenotransplantation for bridging therapy, Anhui Medical University successfully sustained a genetically modified pig liver functioning in a human recipient for 38 days before its removal (8). These outcomes underscore the significant potential of multi-gene modification strategies in overcoming cross-species immune barriers. The BM7G model constructed in this study features a distinct combination and number of genetic modifications, simultaneously targeting two key pathways—complement and coagulation—and theoretically could provide more comprehensive initial protection, offering a new candidate donor for future clinical transplantation. Although the BM7G model shows promising *in-vitro* results, it’s *in-vivo* efficacy and long-term safety require further evaluation. In a preclinical exploratory study, we transplanted a kidney from a BM7G pig into a cynomolgus monkey; the recipient died on postoperative day 27 due to pericardial effusion (potentially related to postoperative management). Although this case was terminated prematurely for non-transplant-related reasons, it still suggests that, beyond the complement and coagulation barriers addressed by our model, xenotransplantation faces complex challenges such as T cell-mediated rejection (53), chronic inflammation (54, 55), and humoral immune responses potentially triggered by non-Gal antibodies (56, 57). These remaining immunological hurdles may require knockout of additional antigens (such as SLA−I/II (58)) or introduction of further immunomodulatory genes (e.g., HO-1 (59), CD39 (27), PD-L1 (60)) to overcome them.

## 5 Conclusion

In a word, we successfully developed a BM7G pig model for xenotransplantation, utilizing endogenous promoters to drive the expression of human protective genes, which ensures more stable expression. The model demonstrated complete elimination of major xenoantigens and synergistic immune-protective functions *in-vitro*. Further pig−to−non−human primate transplantation studies will be essential to evaluate the long−term safety, graft function, and immune rejection of BM7G organs, thereby advancing their clinical transplantation.

## Data Availability

The raw data supporting the conclusions of this article will be made available by the authors, without undue reservation.

## Supplementary material

Supplementary Figure 1 PCR results of the 5’ and 3’ ARM of the knock-in vector at the porcine *Rosa26* locus. The 5’ and 3’ arm bands are 1382 bp and 1614 bp, respectively. Cell clones 6#, 7#, 18#, 19#, 26#, and 27# are positive.

Supplementary Figure 2 Immunofluorescence staining of heart **(A)** and liver **(B)** sections from wild-type (WT, upper panels) and BM7G (lower panels) pigs. The tissues were stained for the expression of three xenoantigens (α-Gal, β-Gal, Neu5Gc) and four Immune compatibility proteins (hCD55, hCD46, hTHBD, hEPCR). The results demonstrate the absence of xenoantigen expression and the successful detection of the human proteins in BM7G tissues. Experiments were independently repeated three times with consistent results. Scale bar = 100 μm.

Supplementary Figure 3 Immunohistochemical (IHC) analysis of heart **(A)** and liver **(B)** sections from wild-type (WT, upper panels) and BM7G (lower panels) pigs. The expression of four immune compatibility proteins (hCD55, hCD46, hTHBD, hEPCR) was detected. Scale bar = 100 μm.

Supplementary Figure 4 Off-Target Analysis for *GGTA1*, *CMAH*, and *B4GalNT2* at the *Rosa26* Locus: Sanger sequencing of WT (top) and TKO (bottom) cells shows identical sequences at predicted off-target sites for *GGTA1* **(A)**, *β4GalNT2* **(B)**, *CMAH* **(C)**, and *Rosa26* **(D)**, indicating no off-target editing.
