# Supplementary figures and images for: Development of a BM7G(TKO/hCD46/hCD55/hTHBD/hEPCR) donor pig with endogenous promoter-driven transgenes for xenotransplantation
Source: Front Immunol. 2026 Jun 23;17:1827497. doi: 10.3389/fimmu.2026.1827497 (PMC13337514; doi:10.3389/fimmu.2026.1827497)

A

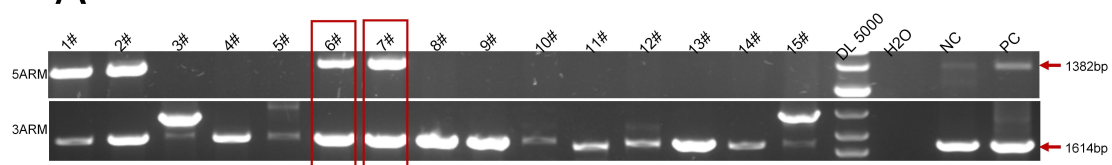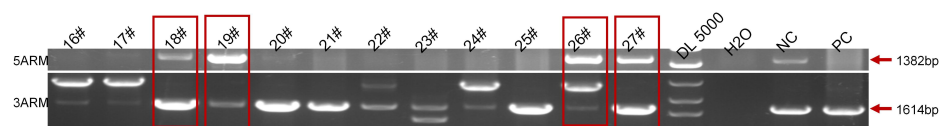

Supplement: Supplementary Figure 1 — PCR results of the 5’ and 3’ ARM of the knock-in vector at the porcine Rosa26 locus. The 5’ and 3’ arm bands are 1382 bp and 1614 bp, respectively. Cell clones 6#, 7#, 18#, 19#, 26#, and 27# are positive. [file Image1.pdf]

A

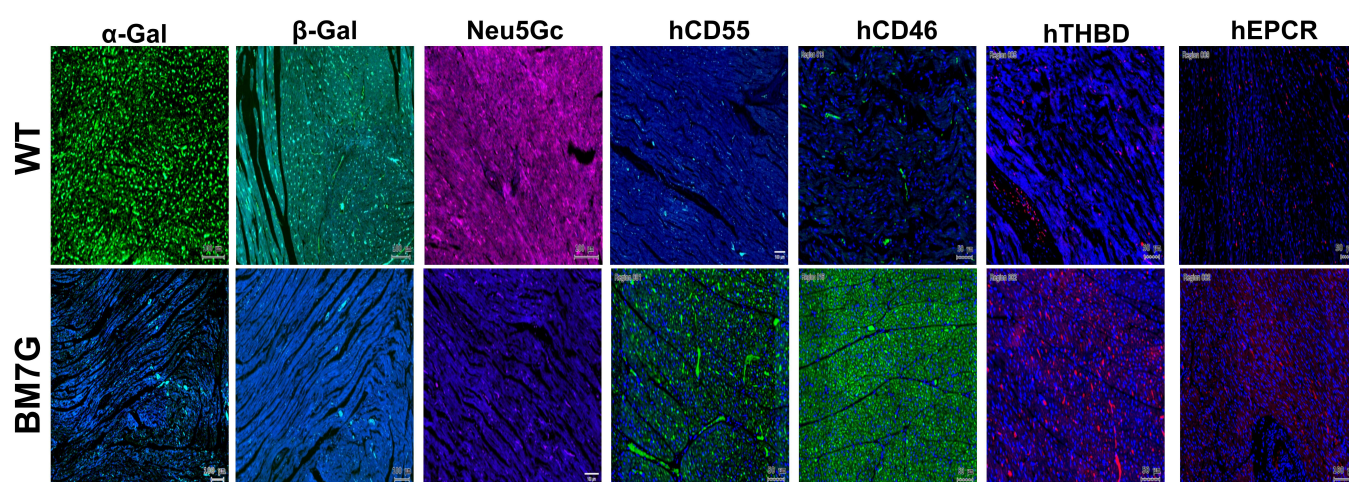

B

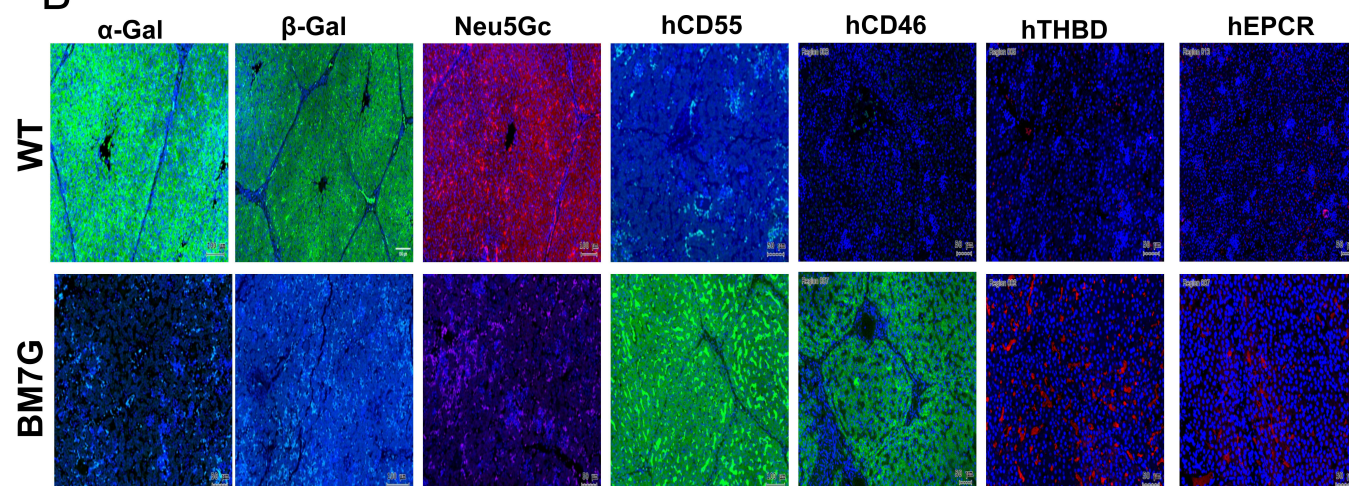

Supplement: Supplementary Figure 2 — Immunofluorescence staining of heart (A) and liver (B) sections from wild-type (WT, upper panels) and BM7G (lower panels) pigs. The tissues were stained for the expression of three xenoantigens (α-Gal, β-Gal, Neu5Gc) and four Immune compatibility proteins (hCD55, hCD46, hTHBD, hEPCR). The results demonstrate the absence of xenoantigen expression and the successful detection of the human proteins in BM7G tissues. Experiments were independently repeated three times with consistent results. Scale bar = 100 μm. [file Image2.pdf]

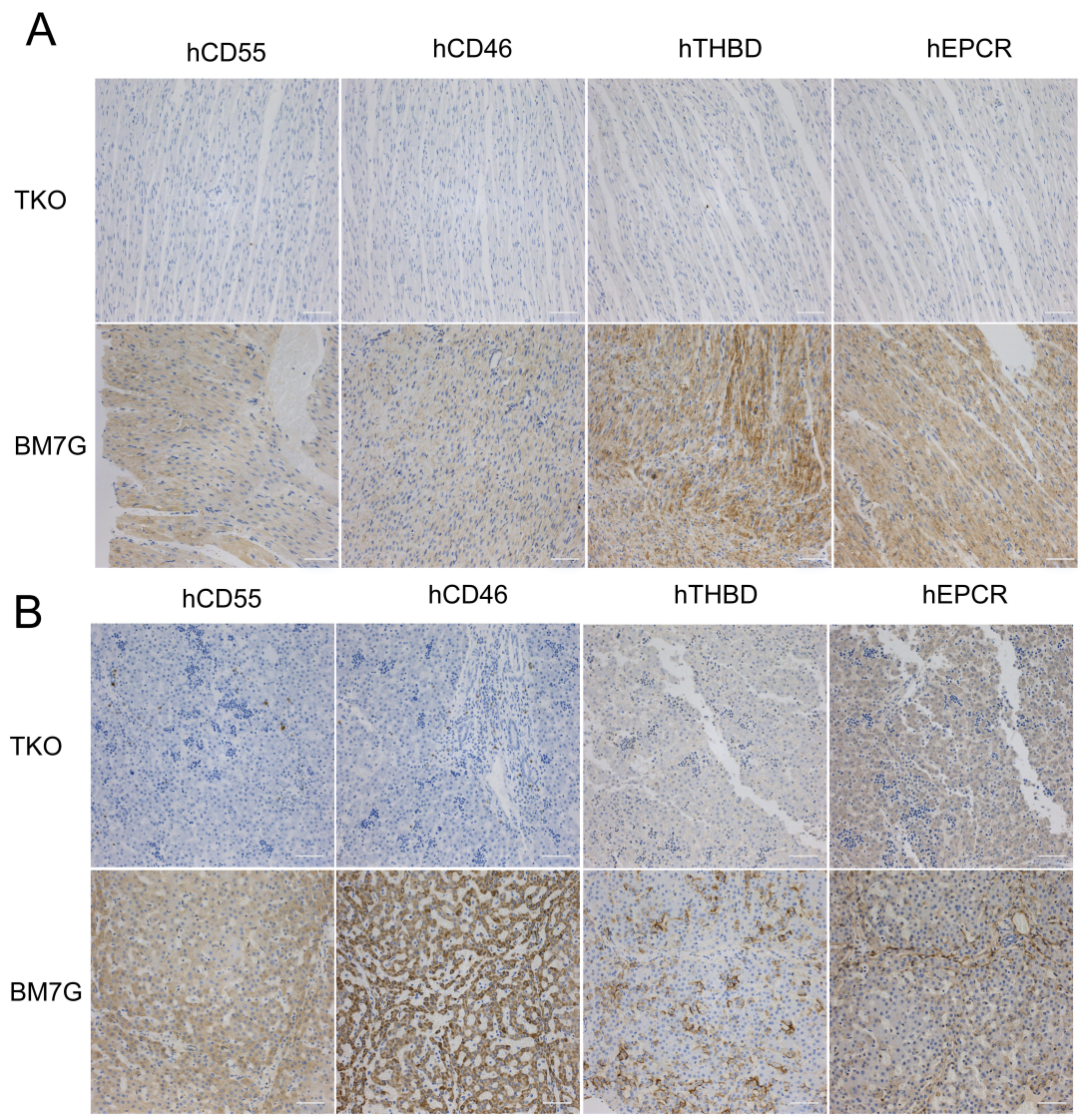

Supplement: Supplementary Figure 3 — Immunohistochemical (IHC) analysis of heart (A) and liver (B) sections from wild-type (WT, upper panels) and BM7G (lower panels) pigs. The expression of four immune compatibility proteins (hCD55, hCD46, hTHBD, hEPCR) was detected. Scale bar = 100 μm. [file Image3.pdf]

## G-OT-1

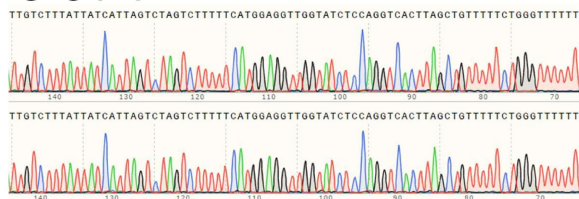

## G-OT-2

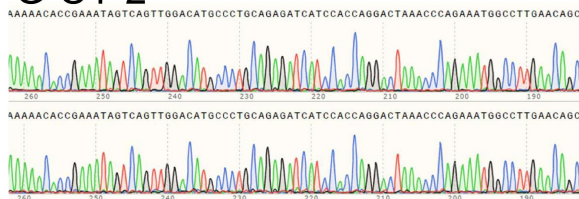

## G-OT-3

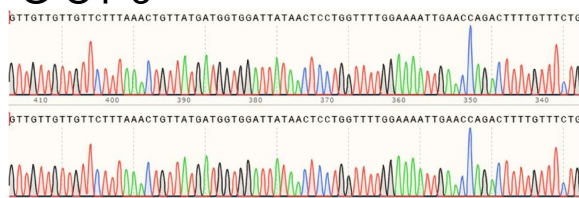

## G-OT-4

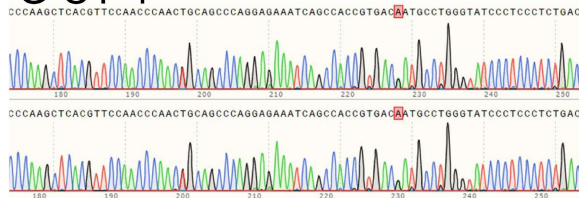

## G-OT-5

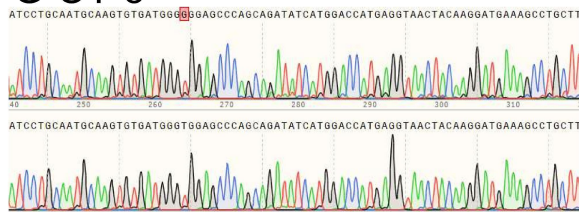

## G-OT-6

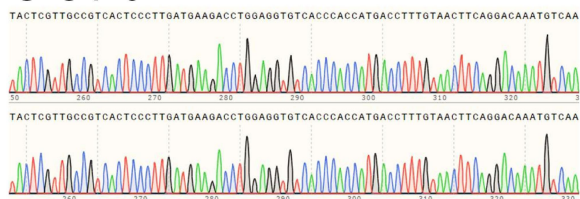

## G-OT-7

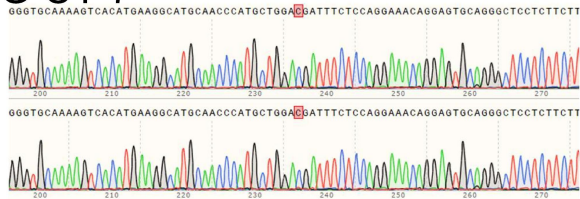

## G-OT-8

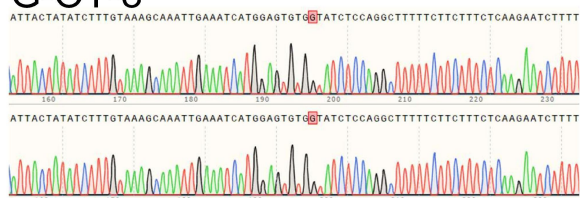

## G-OT-9

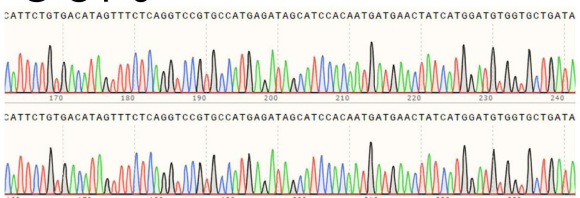

Supplement: Supplementary Figure 4 — Off-Target Analysis for GGTA1, CMAH, and B4GalNT2 at the Rosa26 Locus: Sanger sequencing of WT (top) and TKO (bottom) cells shows identical sequences at predicted off-target sites for GGTA1 (A), β4GalNT2 (B), CMAH (C), and Rosa26 (D), indicating no off-target editing. [file Image4.pdf]

## B-OT-1

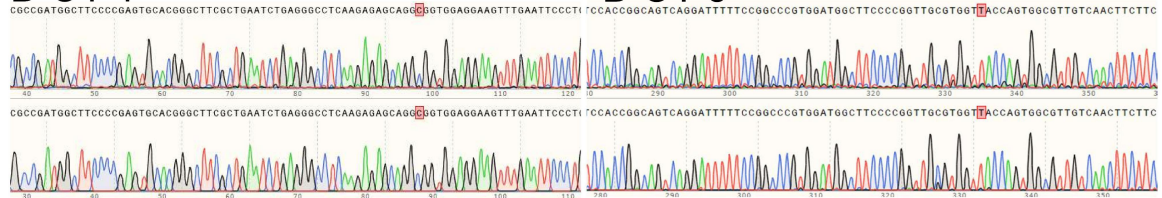

## B-OT-5

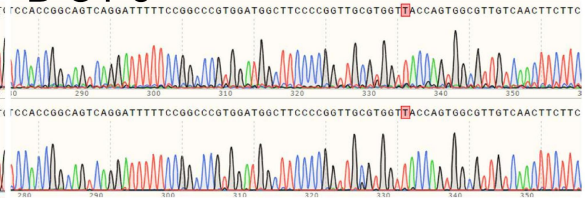

## B-OT-2

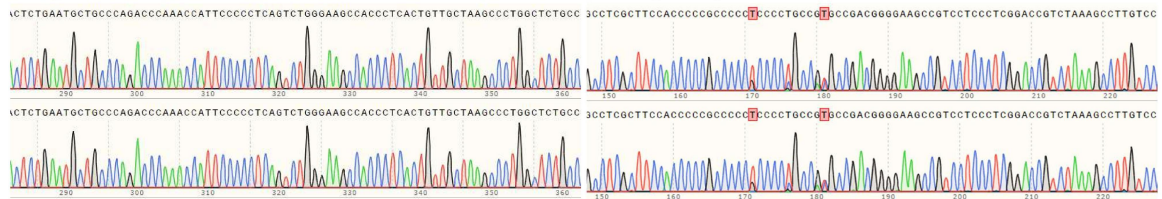

## B-OT-6

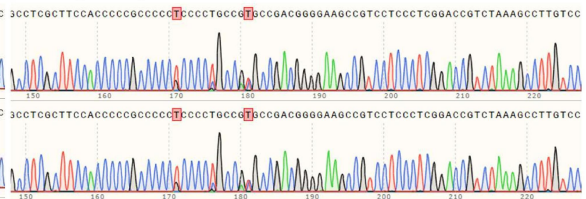

## B-OT-3

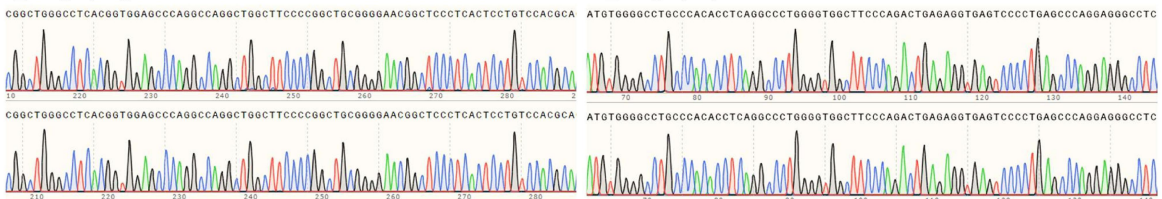

## B-OT-7

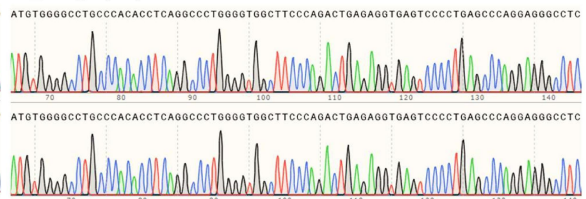

## B-OT-4

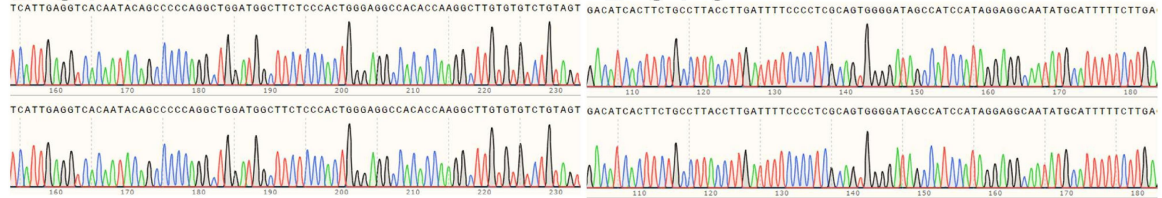

## B-OT-8

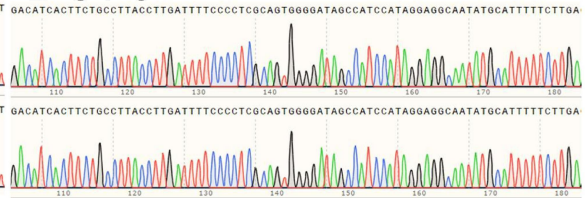

Supplement: Supplementary file 5 [file Image5.pdf]

## C-OT-1

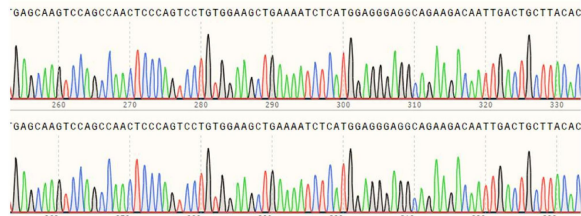

## C-OT-6

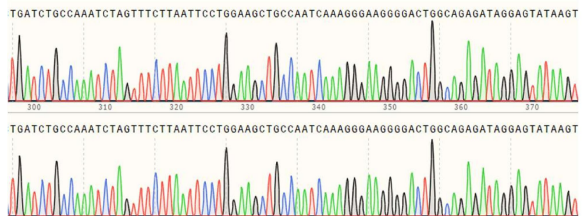

## C-OT-2

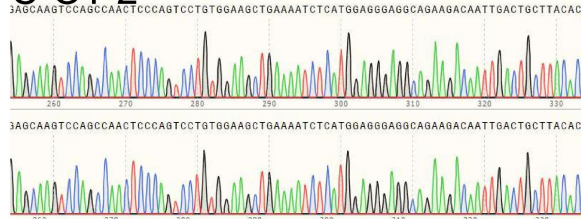

## C-OT-7

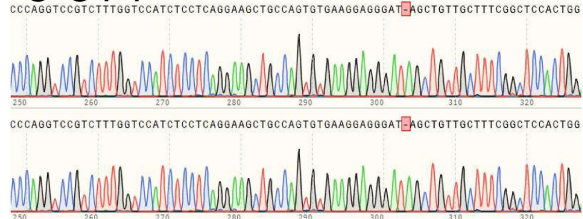

## C-OT-3

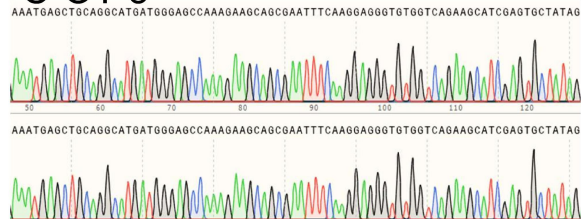

## C-OT-8

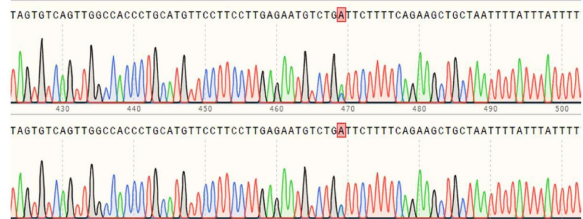

## C-OT-4

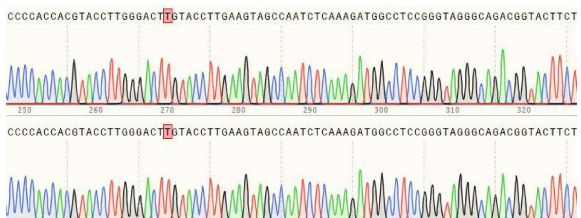

## C-OT-9

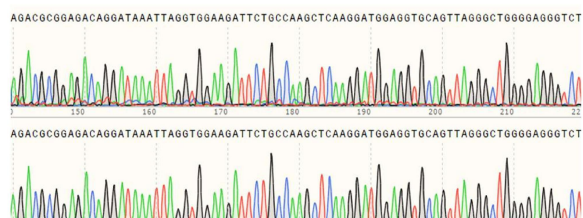

## C-OT-5

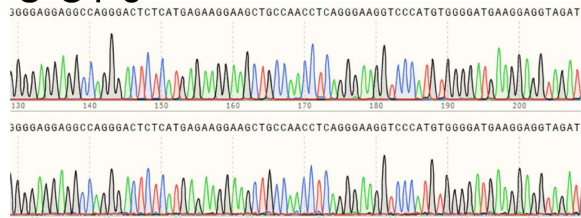

Supplement: Supplementary file 6 [file Image6.pdf]

## R-OT-1

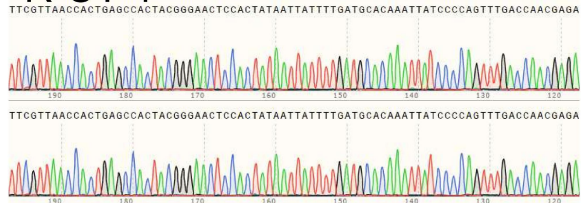

## R-OT-6

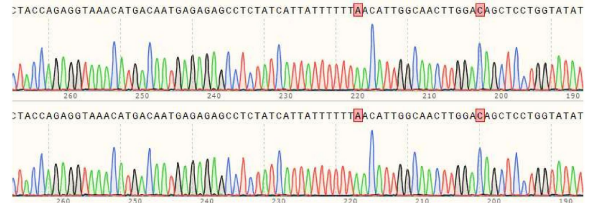

## R-OT-2

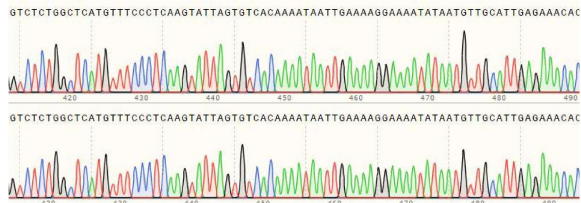

## R-OT-7

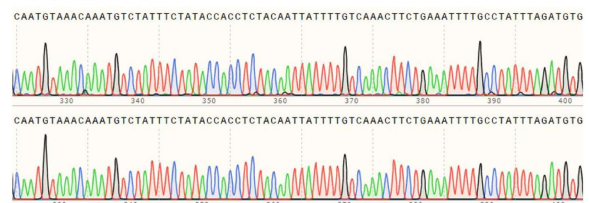

## R-OT-3

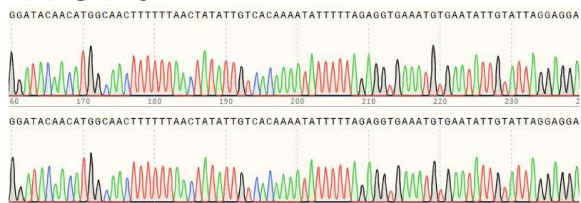

## R-OT-8

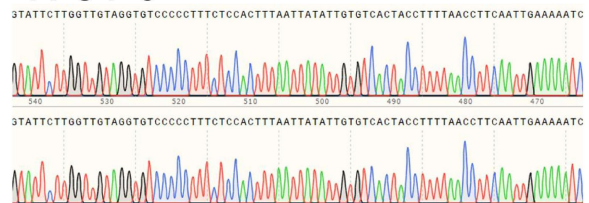

## R-OT-4

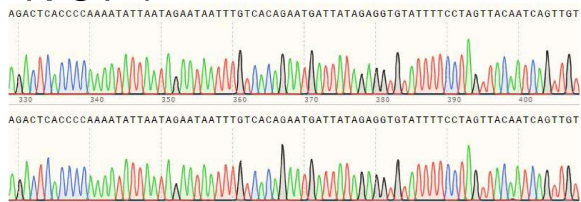

## R-OT-9

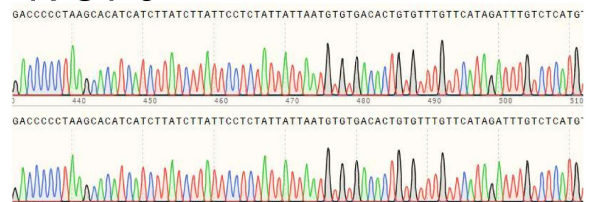

## R-OT-5

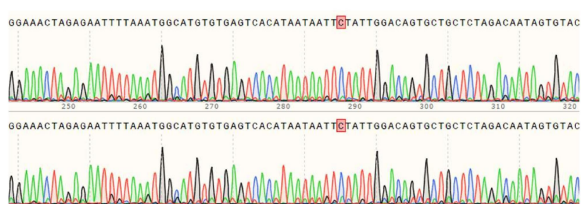

## R-OT-10

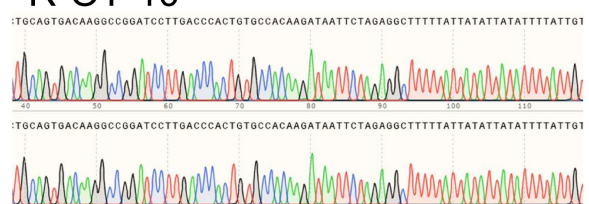

Supplement: Supplementary file 7 [file Image7.pdf]
